# Sepsis Prevalence and Outcome on the General Wards and Emergency Departments in Wales: Results of a Multi-Centre, Observational, Point Prevalence Study

**DOI:** 10.1371/journal.pone.0167230

**Published:** 2016-12-01

**Authors:** Tamas Szakmany, Robert M. Lundin, Ben Sharif, Gemma Ellis, Paul Morgan, Maja Kopczynska, Amrit Dhadda, Charlotte Mann, Danielle Donoghue, Sarah Rollason, Emma Brownlow, Francesca Hill, Grace Carr, Hannah Turley, James Hassall, James Lloyd, Llywela Davies, Michael Atkinson, Molly Jones, Nerys Jones, Rhodri Martin, Yousef Ibrahim, Judith E. Hall, On behalf of Welsh Digital Data Collection Platform Collaborators

**Affiliations:** 1 Department of Anaesthesia, Intensive Care and Pain Medicine, Division of Population Medicine, Cardiff University, Cardiff, United Kingdom; 2 ACT Directorate, Cwm Taf University Health Board, Llantrisant, United Kingdom; 3 Cardiff University Research Society (CUReS), Cardiff University School of Medicine, Cardiff, United Kingdom; 4 Critical Care Directorate, Cardiff and Vale University Health Board, Cardiff, United Kingdom; Lee Kong Chian School of Medicine, SINGAPORE

## Abstract

Data on sepsis prevalence on the general wards is lacking on the UK and in the developed world. We conducted a multicentre, prospective, observational study of the prevalence of patients with sepsis or severe sepsis on the general wards and Emergency Departments (ED) in Wales. During the 24-hour study period all patients with NEWS≥3 were screened for presence of 2 or more SIRS criteria. To be eligible for inclusion, patients had to have a high clinical suspicion of an infection, together with a systemic inflammatory response (sepsis) and evidence of acute organ dysfunction and/or shock (severe sepsis). There were 5317 in-patients in the 24-hour study period. Data were returned on 1198 digital data collection forms on patients with NEWS≥3 of which 87 were removed, leaving 1111 for analysis. 146 patients had sepsis and 144 patients had severe sepsis. Combined prevalence of sepsis and severe sepsis was 5.5% amongst all in-patients. Patients with sepsis had significantly higher NEWS scores (3 IQR 3–4 for non-sepsis and 4 IQR 3–6 for sepsis patients, respectively). Common organ dysfunctions in severe sepsis were hypoxia (47%), hypoperfusion (40%) and acute kidney injury (25%). Mortality at 90 days was 31% with a median (IQR) hospital free stay of 78 (36–85) days. Screening for sepsis, referral to Critical Care and completion of Sepsis 6 bundle was low: 26%, 16% and 12% in the sepsis group. Multivariable logistic regression analysis identified higher National Early Warning Score, diabetes, COPD, heart failure, malignancy and current or previous smoking habits as independent variables suggesting the diagnosis of sepsis. We observed that sepsis is more prevalent in the general ward and ED than previously suggested before and that screening and effective treatment for sepsis and severe sepsis is far from being operationalized in this environment, leading to high 90 days mortality.

## Background

Sepsis is a systemic response to infection, which causes a potentially damaging inflammatory response. Severe sepsis is defined as sepsis leading to dysfunction of one or more organ systems. Successful management of sepsis requires prompt recognition and immediate response with appropriate escalation of care to Critical Care if required [1].

In the UK sepsis is estimated to be responsible for the deaths of 44,000 people every year and to cost the NHS £2.5 billion and for Wales this could equate to a figure of 1800 deaths and a cost of £125 million [2]. However, accurate data collection in the non-Critical Care setting is still under development in Wales and it is thought that the real number is likely to be far higher [3]. Sepsis is a major cause of avoidable mortality and it is imperative that we understand the size of the problem within Wales so that we can improve the quality of care received by our patients.

Through participation in the 1000 Lives Plus RRAILS/Sepsis Wales Programme, all Welsh healthcare organizations have reached a consensus on use of the Sepsis 6 as the optimum treatment protocol with the aim that all elements are delivered within 1 hour of the patient identified as having sepsis [4]. In Wales, one of the cornerstones of identifying sepsis patients is the use of a universally applied track and trigger system, the National Early Warning Score [5]. Its use has been embedded in clinical practice, however it has never been investigated as to whether the preset trigger levels for escalation of care are appropriate for sepsis patients.

Despite evidence demonstrating the value of the Sepsis 6 initiative, marked differences remain between hospitals in the delivery of care for patients with sepsis [4,6,7]. Reviewing the potential barriers to effective application of measures will identify an important opportunity to reduce sepsis-induced mortality further. To inform current and future quality improvement efforts in sepsis, there is a need to better understand how widely and well the evidence-based bundles are used in different hospitals within the same healthcare system. The recent IMPRESS study shed some light on international differences observed in sepsis care in the critical care setting. However there has not been a recent major study of the problem on general wards, the last available data being over 10 years old [8–10].

We have recently reported the results of our point prevalence feasibility study in Wales. They indicated that out of 2716 in-patients in the four hospitals during the 24-hour study period, 51 (1.9%) had signs of infection, classified as sepsis, and 21 (0.8%) had infection and organ dysfunction. Of the 51 patients with sepsis, critical care clinicians saw only seven, of which two patients were admitted to the ICU. Three patients received the full Sepsis 6 bundle within 1 hour [3]. The new sepsis definitions were published in 2016, which potentially has changed the baseline for sepsis incidence, essentially removing the “severe sepsis” category. However our study predated this change and all Welsh hospitals continued to use the 2012 definition of sepsis during the study period.

Based on the lessons learnt from the feasibility pilot we conducted a point-prevalence study across Wales in 2015, utilizing electronic data collection, real-time data monitoring and additional support for the data collectors.

## Methods

### Study design and participants

This was a multicenter, prospective, observational study of the prevalence of patients with either sepsis or severe sepsis on the general wards. On 17 June 2015 (0800 to 0759 hours the following day), consecutive patients presenting to the Emergency Department (ED) or being cared for in an acute in-patient ward setting with sepsis or severe sepsis were enrolled. This date was selected as the medical student data collectors were available during this period and it also represented an “average” day in the Welsh NHS. The data collectors systematically screened every patient on the acute in-patient wards within 4 hours of the study start. The medical student data collectors screened all patients with NEWS≥3 for presence of 2 or more SIRS criteria. To define sepsis, clinical teams had to have a high clinical suspicion of an infection (documented as such in the medical or nursing notes), together with a systemic inflammatory response (sepsis) and evidence of acute organ dysfunction and/or shock (severe sepsis) [1]. Patients were excluded if they were less than 18 years of age or if they were in a Critical Care environment. Participating hospitals were identified through local collaborators via the Welsh Intensive Care Society Audit and Research Group. The project was approved by the West Midlands Regional Ethics Committee (15/WM/0095) and patients gave written informed consent. All demographic and clinical information were de-identified as part of data collection processes so that patient anonymity was strictly maintained throughout the study. The Size of Sepsis in Wales project was registered with an international trial registry (ISRCTN78293101).

Local investigators were identified and were supported by three national coordinators. Key study information was provided through e-mails, face-to-face training and online video tutorials, which included the protocol, answers to key questions and description of the electronic case report form (eCRF). The details of the digital data collection platform developed for this study have been published previously [11]. Medical students working in pairs to ensure data validity and appropriate clinical knowledge, acted as data collectors, using tablets for electronic data collection and transfer. The tablets contained all supporting information needed for the study, including national formulary. Data collectors were supported by continuous online web-chat, which made the senior clinicians and the medical student national coordinators available throughout the study period. Upon entry into the eCRF, each patient was assigned a unique study identifier. No patient identifiable data was submitted to the online database. The data collected were all part of routine clinical care. Patients were followed up until 90 days after study enrolment.

Data were collected for every patient, on whether their management fulfilled the requirements of the Sepsis 6 bundle.

### Statistical analysis

Categorical variables are described as proportions and are compared using chi-square or Fisher’s exact test. Continuous variables are described as mean and standard deviation if normally distributed or median and inter-quartile range if not normally distributed. Comparisons of continuous variables are performed using one-way ANOVA or Mann-Whitney test as appropriate. To assess the baseline factors associated with development of sepsis, a multi-variable logistic regression analysis was performed. Comorbidities, demographic and medication-related variables were entered using forced simultaneous entry. A process of forward and backward selection, based on minimisation of Akaike’s An Information Criterion (AIC), was used to derive the final model. Results are presented as odds ratios with 95% confidence intervals. Significance is set at p<0.05. A final analysis was performed at the end of the study.

## Results

**Fig 1:**
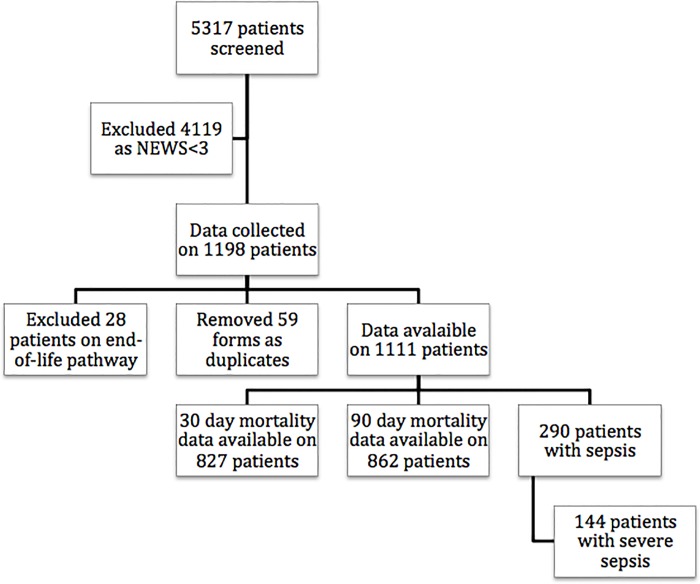
Organisational flowchart of the study

We collected data to measure the point-prevalence of sepsis and severe sepsis in 15 hospitals in Wales over a 24-hour period. This included all hospitals where 24/7 Consultant led ED was operational, with ability to admit and treat any acutely unwell patient, ranging from tertiary academic centers with almost 1000 inpatient beds to small local district general hospitals with 130 inpatient beds (S1 Table). There were 5317 in-patients in the 24-hour study period. Data were returned on 1198 digital data collection forms on patients with NEWS≥3 of which 87 were removed having been identified as duplicates (n = 59) or where patients were clearly on an end-of-life pathway (n = 28), leaving 1111 for analysis (Fig 1). From the 1111 “at risk” patients 208 was admitted to ED and 903 were on the general wards. Out of these patients 146 had sepsis and 144 had severe sepsis according to the Surviving Sepsis Campaign definition (35 sepsis and 42 severe sepsis in ED; 111 sepsis and 102 severe sepsis on the wards, respectively). Patients with sepsis had significantly higher NEWS scores, had significantly more chronic health problems and were more likely to be treated with long-term steroids compared non-sepsis patients (Table 1). At 90 days significantly more patients in the sepsis group had died (31.5% vs. 23.3%, respectively). Patients suffering from sepsis had also significantly less hospital free days at 90-days compared to non-sepsis patients (Table 2).

**Table 1:** Basic demographic data of sepsis and non-sepsis patients

	Sepsis patients (n = 290)	Non-sepsis patients (n = 821)	p-value
**Age**	75 (61–84)	75 (63–83)	0.937
**Male/female**	141/149 (48.6%/51.4%)	376/445 (45.8%/54.2%)	0.408
**National Early Warning Score**	4 (3–6)	3 (3–4)	<0.001
**Admission to the hospital**			
**Home**	252 (86.9%)	729 (88.8%)	0.528
**Nursing home**	10 (3.4%)	36 (4.4%)	
**Supported accommodation**	9 (3.1%)	19 (2.3%)	
**Other hospital**	12 (4.1%)	23 (2.8%)	
**Other**	7 (2.4%)	14 (1.7%)	
**Medical history**			
**Alcohol abuse**	22 (7.6%)	12 (1.5%)	<0.001
**Smoker**	39 (13.4%)	11 (1.3%)	<0.001
**Ex-smoker**	57 (19.7%)	17 (2.1%)	<0.001
**COPD**	74 (25.5%)	23 (2.8%)	<0.001
**Diabetes**	77 (26.6%)	18 (2.2%)	<0.001
**Heart failure**	39 (13.4%)	13 (1.6%)	<0.001
**Renal failure**	24 (8.3%)	8 (1.0%)	<0.001
**Malignancy**	56 (19.3%)	12 (1.5%)	<0.001
**Medication history**			
**ACE inhibitors**	72 (24.8%)	195 (23.8%)	0.713
**Beta-blockers**	83 (28.6%)	229 (27.9%)	0.813
**Diuretics**	101 (34.8%)	265 (32.3%)	0.428
**Statins**	92 (31.7%)	282 (34.3%)	0.415
**Insulin**	23 (7.9%)	39 (4.8%)	0.05
**Immunosuppressant**	12 (4.1%)	24 (2.9%)	0.328
**Chronic oral steroids**	63 (21.7%)	110 (13.4%)	<0.001
**Chronic antibiotics**	15 (5.2%)	27 (3.3%)	0.161

Data presented in percentages or median (interquartile range). For statistical analysis Fisher’s exact test or Mann-Whitney U test was used as appropriate. Other admission source: temporary residence such as holiday home

**Table 2:** Patient outcomes in sepsis and non-sepsis groups

	Sepsis (n = 290)	Non-sepsis (n = 821)	p-value
**Mortality at 30 days (%)**	47 (22.0%)	88 (14.3%)	0.001
**Mortality at 90 days (%)**	70 (31.5%)	149 (23.3%)	0.017
**Hospital free days at 90 days**	74 (0–85)	80 (47–87)	0.011

Data presented in percentages or median (interquartile range). For statistical analysis Fisher’s exact test or Mann-Whitney U test was used as appropriate. Mortality data was missing at day 30 in 77 sepsis and 207 non-sepsis patients, at day 90 in 68 sepsis and 181 non-sepsis patients

In the multivariable logistic regression model, National Early Warning Score, history of diabetes, COPD, heart failure, malignancy and current or previous smoking habits were identified as independent variables suggesting the diagnosis of sepsis (Table 3).

**Table 3:** Results of logistic regression model for diagnosing sepsis

	OR (95% CI)	p-value
**NEWS**	1.64 (1.46–1.84)	<0.001
**COPD**	3.88 (2.03–7.04)	<0.001
**Heart failure**	3.42 (1.52–7.72)	<0.001
**Diabetes**	13.03 (7.09–23.93)	<0.001
**Malignancy**	13.93 (6.81–28.48)	<0.001
**Ex-smoker**	3.82 (1.87–7.82)	<0.001
**Smoker**	8.18 (3.58–18.68)	<0.001

Data presented as adjusted odds ratios presented with 95% confidence intervals.

We compared the sepsis and severe sepsis groups, to understand whether there were any significant differences between patients who go on to develop the more severe form of the condition. Table 4 shows the baseline demographic data, care metrics and outcomes. Overall, 50% of the patients with sepsis or severe sepsis were male and 25% presented with at least one co-morbid illness. Chronic, regular medications were used during the study period in 67% and 80% of sepsis and severe sepsis patients, respectively. The majority of patients were diagnosed with sepsis on the ward (73%) and the most frequent presentations were medical admissions (72%). The most common organ dysfunction at presentation were hypoxia (47%), hypoperfusion (40%) and acute kidney injury (25%). The overall mortality at 90 days was 31% with a median (IQR) hospital free days of 78 (36–85). Amongst the patients identified as suffering from sepsis, structured screening for the condition, referral to Critical Care and completion of the Sepsis 6 bundle was low at 26%, 16% and 12% respectively.

**Table 4:** Comparison of patients with sepsis and severe sepsis

	Sepsis (n = 146)	Severe sepsis n = 144)	p-value
**Age (years)**	74 (57–83)	75 (64–84)	0.106
**Male sex**	66 (45%)	75 (52%)	
**NEWS**	4 (3–5)	5 (4–7)	<0.001
**Presenting with chronic conditions**	92 (63%)	117 (81%)	0.001
**Admission from home**	130 (89%)	122 (85%)	0.30
**Patient location at time of study screening**			
**ED**	35 (24%)	43 (30%)	0.29
**Ward**	111 (76%)	101 (70%)	0.29
**Source of infection**			
**Pulmonary**	76 (52%)	91 (63%)	0.06
**Abdominal**	45 (30%)	41 (29%)	0.7
**Urinary**	16 (11%)	16 (11%)	0.99
**Other**	10 (7%)	9 (6%)	0.8
**Care metrics and outcome**			
**Seen by Critical Care team**	9 (6%)	15 (10%)	0.15
**Sepsis 6 completed**	4 (3%)	13 (9%)	0.03
**Screening tool completed**	14 (10%)	23 (16%)	0.12
**90-day mortality**	42 (29%)	49 (34%)	0.38

Data presented in percentages or median (interquartile range). For statistical analysis Fisher’s exact test or Mann-Whitney U test was used as appropriate. Mortality data was missing at day 30 in 77 patients, at day 90 in 68 patients. Other infection source: skin, soft tissue or wound infection.

Certain elements of the Sepsis 6 were completed, however even compliance with individual elements was below the expected level of 80% (Tables 5 and 6).

**Table 5:** Sepsis 6 bundle elements completed in sepsis and severe sepsis patients

	O2 administration	Lactate measurements	Blood culture	i.v. antibiotic administration	Fluid bolus	Fluid balance
**Sepsis n = 146**	42 (29%)	30 (21%)	25 (17%)	59 (40%)	32 (22%)	56 (38%)
**Severe sepsis n = 144**	87 (60%)	55 (38%)	43 (30%)	77 (54%)	49 (34%)	65 (45%)

Data presented in percentages.

**Table 6:** Sepsis 6 bundle completion in sepsis and severe sepsis patients by number of elements

Bundle completion	1 element	2 elements	3 elements	4 elements	5 elements	Full bundle	None
**Sepsis n = 146**	39 (27%)	17 (12%)	20 (14%)	8 (6%)	11 (8%)	4 (3%)	47 (32%)
**Severe sepsis n = 144**	23 (16%)	30 (21%)	21 (15%)	23 (16%)	12 (8%)	13 (9%)	22 (15%)

Data presented in percentages.

Thirty-two (22%) of patients in the sepsis group and 46 (32%) of patients in the severe sepsis group had NEWS ≥6, which is the recognized trigger for escalation of care towards senior medical review and critical care support. Moreover, 4 (3%) of sepsis patients and 15 (11%) of the severe sepsis patients had NEWS ≥9, which would mandate immediate critical care review. None of the sepsis patients and 5 out of 15 of the severe sepsis patients with NEWS ≥9 were seen by Critical Care Teams during the study period.

Survivors in the sepsis group tended to be younger 73 (58–82) vs. 83 (74–87) years and had more hospital free days 82 (66–86) vs. 0 (0–58) days survivors and non-survivors, p<0.001 respectively.

We found no significant differences between survivors and non-survivors with respect to male to female ratio, distribution of background medical and lifestyle problems or in the rate of delivery of individual components of the Sepsis 6 bundle.

## Discussion

In our multicenter study we found that 5.5% of all in-patients had sepsis and half of these had severe sepsis. This is higher than the 4.4% incidence found in the Esteban study in 2003 and reflects the global increase in the disease burden found in recent analysis of hospital and ICU registries [10,12]. To our knowledge, this is the first study in the UK to measure the prevalence of sepsis and severe sepsis in the non-critical care setting, using contemporary data collection methods and utilizing the SCCM Consensus criteria [1]. The 90-day mortality of sepsis and severe sepsis was 31%. The recent IMPRESS study, which only looked at patients cared for in the ED and on the ICU, found that severe sepsis in Western Europe has mortality in the region of 26%, whereas in our series in patients admitted to the general wards was 34% [8]. The observed mortality is in-line with those from the Surviving Sepsis Campaign observational studies, but significantly higher than the recently completed interventional RCTs in severe sepsis [13–17]. These interventional trials examining early goal-directed therapy may recruit a different patient population and they also offer significantly greater protocolised care, than observational studies [8]. Interestingly, none of our participating hospitals were involved in the UK based PROMISE study, hence our results could be generalized to the majority of hospitals, who have not developed protocolised sepsis pathways as part of an externally driven randomized trial [17].

Despite widespread support in the use of the Sepsis Screening Tool and Sepsis 6 bundle in Wales, our results suggest that adoption of these evidence based practices is still low: only 9% of the patients had screening tool completed and only 5.8% had the full Sepsis 6 bundle. These results confirm our previous findings in the feasibility pilot [3]. There are many possible reasons for this disappointing result. Although sepsis awareness has been raised in recent years amongst all hospital practitioners, too often it is still perceived as a primarily “critical care problem”. Hospital practitioners are encouraged to think about sepsis if a patient scores 3 or higher on the NEWS chart. However, the nationwide track and trigger system only calls for senior review and critical care involvement when a patient scores 6 or higher [7]. Our data clearly indicates that more than half of sepsis patients had low NEWS and this apparent lack of physiological deterioration could have led to sepsis being overlooked. Our methods allowed us to strictly follow the guidance for sepsis screening and this increased scrutiny is probably responsible for many of the sepsis cases discovered. As detection of the syndrome was low during usual clinical care, it is unsurprising, that the Sepsis 6 bundle has only been applied in ad-hoc manner [3]. It has been shown previously that improving the detection, raising awareness of the problem and concentrated efforts to improve basic sepsis care can lead to significant mortality reduction, even if bundle compliance remains low [18]. Possibly due to the low sample size, we could not show any mortality benefit from use of the full Sepsis 6 bundle, or any individual elements applied.

Our data adds further support to the argument that the diagnosis of sepsis is in need of revision [19]. Recently, the ANZICS group has demonstrated that “SIRS negative” sepsis is prevalent and has similarly high mortality as “SIRS positive” sepsis [20]. As individual elements of the NEWS are very similar to some of the SIRS criteria (i.e. temperature, respiratory rate, heart rate, altered level of consciousness) it is plausible that some of the “SIRS negative” patients, especially with higher NEWS (n = 52 when NEWS ≥6) were indeed suffering from sepsis or severe sepsis. This would need further confirmation in another study employing methods to capture sepsis prevalence according to both the 2012 Consensus definition and the new Sepsis 3.0 clinical criteria [21,22].

The multivariate regression model also suggested that higher NEWS is significantly associated with the diagnosis of sepsis. With this information in mind a recalibration of the sepsis diagnosis and treatment pathway might be appropriate, as almost half of the patients suffering from sepsis induced acute organ dysfunction had low NEWS, which would not have prompted senior review. Perhaps this lack of medical and nursing involvement may explain the low compliance with the Sepsis 6 bundle, in particular the low rate of antibiotic administration. It has been shown previously that track and trigger scoring systems and NEWS in particular is only moderately effective in predicting clinical deterioration, ICU admission or death in sepsis patients in the ED and on the general wards [23,24]. Our results further suggest, that robust screening regardless of the actual NEWS value is necessary to be able to measure the burden of sepsis. Although Critical Care Outreach and ED teams have been cornerstone of raising the profile of sepsis care in the hospitals, further education of frontline ward staff is needed to improve the detection and effective treatment of sepsis.

The strengths of our study include the innovative data collection of the defined dataset, the rigorous training of the data collectors via multimedia platforms and the wide participation of the Welsh hospitals. We screened all patients in every participating hospital during the study period, thus gaining a complete picture of the problem. We have described a cohort of patients with sepsis and severe sepsis, who can be identified in EDs and general wards in the UK and in Western Europe. We have previously described our methods in our feasibility study and the evidence based bundle elements have been implemented in all participating centers through the 1000 Lives quality improvement initiative [3,7]. We were then able to collect data describing compliance with these metrics and also data describing presentation patterns and severity of these patients.

Our study has some limitations. Our data set was a compromise between being an exhaustive list describing all facets of a patient with sepsis and being small enough to encourage site participation and data reliability. This ‘point’ estimate reduces the external validity, as there is likely to be significant variance in both admission numbers of patients presenting to hospital and clinical practice on a day-to-day basis and also does not compensate for known seasonal variations in incidence of the condition. This may therefore not reflect the true picture of sepsis mortality in Welsh acute hospitals over time. However, in the absence of more robust data on the Size of the Sepsis problem, it represents the best estimate available. In addition, we followed our patients up for only 90 days; consequently we have little understanding as to what happened to the patients following that period and their quality of life following discharge.

It is possible that a proportion of our patients who did not fulfill 2 or more SIRS criteria were in fact suffering from sepsis [20–22]. Unfortunately, due to the data collection algorithm employed, we were unable to quantify this in our dataset. We could also have missed patients with sepsis, who had NEWS below 3 (e.g. patients high temperature and white cell count, but normal respiratory rate and heart rate). Despite the fact that NEWS scores disproportionately highly for patients with respiratory pathology, our distribution of source of infection is close to that of other previously published studies, suggesting that our screening methods did not bias for or against an important subgroup of patients [4,8].

There is a lot more to do in understanding the burden of sepsis on the general wards and we plan to use the recently established SAIL database to gather more information about resource utilization and long-term outcome of patients suffering from sepsis, regardless of the definition used [25]. Chronic comorbidities and the frequency of regular medications, especially the use of chronic, oral corticosteroid treatment, could reflect un-modifiable patient characteristics in sepsis and severe sepsis. The impact of these on any specific treatment option for the condition needs further investigation.

Our study has confirmed our previous report and that of others, that compliance with Sepsis 6 bundles and SCC resuscitation bundles is poor if it is not targeted by a dedicated “Sepsis Team” [3,4,8]. This is the first study to report compliance with Sepsis 6 bundles in an unselected population. Bundle compliance was similar between the participating centers, confirming this is a system issue and not an isolated problem. In contrast to the work of others, we cannot confirm that compliance with the Sepsis 6 bundle has an effect on outcome, however our sample size was very small [4,6].

In conclusion, we observed that sepsis is more prevalent in the general ward and ED than previously suggested before and that screening and effective treatment for sepsis and severe sepsis is far from being operationalized in this environment, leading to high 90 days mortality.

## Supporting Information

S1 Table Characteristics of the participating hospitals (DOCX)

## References

[1] Levy MM, Fink MP, Marshall JC, Abraham E, Angus D, Cook D et al 2001 SCCM/ESICM/ACCP/ATS/SIS International Sepsis Definitions Conference. Crit Care Med. 2003:530–538.

[2] Shahin J, Harrison DA, Rowan KM. Relation between volume and outcome for patients with severe sepsis in United Kingdom: retrospective cohort study. BMJ. 2012;344(may29 1):e3394–e3394. 22645208 10.1136/bmj.e3394 PMC3362472

[3] Szakmany T, Ellis G, Lundin RM, Pignatelli I, Sharif B, Joshi S et al Sepsis in Wales on the general wards: results of a feasibility pilot. British Journal of Anaesthesia. 2015;114(6):1000–1001. 10.1093/bja/aev133 25991742

[4] Daniels R, Nutbeam T, McNamara G, Galvin C. The sepsis six and the severe sepsis resuscitation bundle: a prospective observational cohort study. Emerg Med J. 2011;28(6):507–512. 10.1136/emj.2010.095067 21036796

[5] McGinley A, Pearse RM. A national early warning score for acutely ill patients. BMJ. 2012;345(aug08 1):e5310–e5310. 22875955 10.1136/bmj.e5310

[6] Szakmany T, Burke J, Smith L, Hermon A. 947. Crit Care Med. 2014;42:A1588.

[7] Hancock C. A national quality improvement initiative for reducing harm and death from sepsis in Wales. Intensive Crit Care Nurs. 2015;31(2):100–105. 10.1016/j.iccn.2014.11.004 25604031

[8] Rhodes A, Phillips G, Beale R, Cecconi M, Chiche JD, De Backer D et al The Surviving Sepsis Campaign bundles and outcome: results from the International Multicentre Prevalence Study on Sepsis (the IMPreSS study). Intensive Care Med. 2015;41(9):1620–1628. 10.1007/s00134-015-3906-y 26109396

[9] Szakmany T, Ellis G, Sharif B, Lundin RM, Hall JE. Preventing sepsis. Lancet Infect Dis. 2015;15(11):1260. 10.1016/S1473-3099(15)00376-X 26531032

[10] Esteban A, Frutos-Vivar F, Ferguson ND, Penuelas O, Lorente JA, Gordo F et al Sepsis incidence and outcome: contrasting the intensive care unit with the hospital ward. Crit Care Med. 2007;35(5):1284–1289. 10.1097/01.CCM.0000260960.94300.DE 17414725

[11] Sharif B, Lundin RM, Morgan P, Hall JE, Dhadda A, Mann C et al Developing a digital data collection platform to measure the prevalence of sepsis in Wales. J Am Med Inform Assoc. 2016 4 19. pii: ocv208. 10.1093/jamia/ocv208 PMC11960762 27094989

[12] Rhee C, Murphy MV, Li L, Platt R, Klompas M, Centers for Disease Control and Prevention Epicenters Program. Comparison of trends in sepsis incidence and coding using administrative claims versus objective clinical data. Clin Infect Dis. 2015;60(1):88–95. 10.1093/cid/ciu750 25258352 PMC4318944

[13] Levy MM, Artigas A, Phillips GS, Rhodes A, Beale R, Osborn TM et al Outcomes of the Surviving Sepsis Campaign in intensive care units in the USA and Europe: a prospective cohort study. Lancet Infect Dis. 2012;12(12):919–924. 10.1016/S1473-3099(12)70239-6 23103175

[14] Levy MM, Rhodes A, Phillips GS, Townsend SM, Schorr CA, Beale R et al Surviving Sepsis Campaign: association between performance metrics and outcomes in a 7.5-year study. Intensive Care Med. 2014;40(11):1623–1633. 10.1007/s00134-014-3496-0 25270221

[15] ProCESS Investigators. A randomized trial of protocol-based care for early septic shock. N Engl J Med. 2014;370(18):1683–1693. 10.1056/NEJMoa1401602 24635773 PMC4101700

[16] ARISE Investigators and the ANZICS Clinical Trials Group. Goal-directed resuscitation for patients with early septic shock. N Engl J Med. 2014;371(16):1496–1506. 10.1056/NEJMoa1404380 25272316

[17] Mouncey PR, Osborn TM, Power GS, Harrison DA, Sadique MZ, Grieve RD et al Trial of early, goal-directed resuscitation for septic shock. N Engl J Med. 2015;372(14):1301–1311. 10.1056/NEJMoa1500896 25776532

[18] Westphal GA, Koenig Á, Caldeira Filho M, Feijo J, de Oliveira LT et al Reduced mortality after the implementation of a protocol for the early detection of severe sepsis. J Crit Care. 2011;26(1):76–81. 10.1016/j.jcrc.2010.08.001 21036531

[19] Cohen J, Vincent J-L, Adhikari NKJ, Machado FR, Angus DC, Calandra T et al Sepsis: a roadmap for future research. Lancet Infect Dis. 2015;15(5):581–614. 10.1016/S1473-3099(15)70112-X 25932591

[20] Kaukonen K-M, Bailey M, Pilcher D, Cooper DJ, Bellomo R. Systemic inflammatory response syndrome criteria in defining severe sepsis. N Engl J Med. 2015;372(17):1629–1638. 10.1056/NEJMoa1415236 25776936

[21] Seymour CW, Liu VX, Iwashyna TJ, Brunkhorst FM, Rea TD, Scherag A et al Assessment of Clinical Criteria for Sepsis: For the Third International Consensus Definitions for Sepsis and Septic Shock (Sepsis-3). JAMA. 2016;315(8):762–774. 10.1001/jama.2016.0288 26903335 PMC5433435

[22] Singer M, Deutschman CS, Seymour CW, Shankar-Hari M, Annane D, Bauer M et al The Third International Consensus Definitions for Sepsis and Septic Shock (Sepsis-3). JAMA. 2016;315(8):801–810. 10.1001/jama.2016.0287 26903338 PMC4968574

[23] Yu S, Leung S, Heo M, Soto GJ, Shah RT, Gunda S et al Comparison of risk prediction scoring systems for ward patients: a retrospective nested case-control study. Crit Care. 2014;18(3):R132 10.1186/cc13947 24970344 PMC4227284

[24] Corfield AR, Lees F, Zealley I, Houston D, Dickie S, Ward K et al Utility of a single early warning score in patients with sepsis in the emergency department. Emerg Med J. 2014;31(6):482–487. 10.1136/emermed-2012-202186 23475607

[25] Jones KH, Ford DV, Jones C, Dsilva R, Thompson S, Brooks CJ et al A case study of the Secure Anonymous Information Linkage (SAIL) Gateway: a privacy-protecting remote access system for health-related research and evaluation. J Biomed Inform. 2014;50:196–204. 10.1016/j.jbi.2014.01.003 24440148 PMC4139270

