# Supplementary material for: Sepsis Prevalence and Outcome on the General Wards and Emergency Departments in Wales: Results of a Multi-Centre, Observational, Point Prevalence Study
Source: PLoS One. 2016 Dec 1;11(12):e0167230. doi: 10.1371/journal.pone.0167230 (PMC5132245; doi:10.1371/journal.pone.0167230)
Supplement: S1 Table — (DOCX) [file pone.0167230.s001.docx]

**S1 Table Characteristics of the participating hospitals:**

Hospital A: Large district general hospital with 774 inpatient beds, 16 critical care beds

Hospital B: Medium district general hospital with 382 inpatient beds, 8 critical care beds

Hospital C: Tertiary academic centre with 954 inpatient beds, 32 critical care beds

Hospital D: Medium district general hospital with 429 inpatient beds, 6 critical care beds

Hospital E: Medium district general hospital with 453 inpatient beds, 10 critical care beds

Hospital F: Medium district general hospital with 388 inpatient beds, 6 critical care beds

Hospital G: Medium district general hospital with 435 inpatient beds, 8 critical care beds

Hospital H: Tertiary academic centre with 729 inpatient beds, 24 critical care beds

Hospital I: Medium district general hospital with 359 inpatient beds, 14 critical care beds

Hospital J: Small district general hospital with 205 inpatient beds, 5 critical care beds

Hospital K: Small district general hospital with 231 inpatient beds, 7 critical care beds

Hospital L: Small district general hospital with 139 inpatient beds, 3 critical care beds

Hospital M: Medium district general hospital with 452 inpatient beds, 11 critical care beds

Hospital N: Medium district general hospital with 484 inpatient beds, 11 critical care beds

Hospital O: Medium district general hospital with 602 inpatient beds, 12 critical care beds
